# Supplementary material for: Are carbon emissions trading and green financial instruments synergistic? -Comprehensive quantitative research based on content analysis
Source: PLoS One. 2024 Mar 7;19(3):e0298601. doi: 10.1371/journal.pone.0298601 (PMC10919601; doi:10.1371/journal.pone.0298601)
Supplement: S1 Data — (ZIP) [file pone.0298601.s001.zip › Data/地级市Z_F工作报告.zip!/╕╩╦α/└╝╓▌╩╨/└┤╫╘╣1⁄2╓┌║┼í╛┬φ┐╦╩2╛▌═°í┐_2010.docx]

各位代表：

　　现在，我代表市人民政府向大会作工作报告，请予审议，并请各位政协委员和其他列席人员提出意见。

　　一、2009年政府工作回顾

　　刚刚过去的2009年，我们在省委、省政府和市委的正确领导下，以科学发展观统领经济社会发展全局，认真落实国家扩大内需的政策措施，积极应对全球金融危机的冲击和影响，按照“1355”总体发展思路，突出保增长、保项目、保民生、保节能减排、保稳定的工作重点，强化工作措施，全力推动发展，完成了市人大十四届四次会议确定的各项目标任务。

　　(一)全市经济平稳较快发展。预计实现生产总值920亿元，增长10．5%以上；完成全社会固定资产投资506.18亿元，增长17.18%。加强工业经济运行调控服务，出台加快推进新型工业化及六大产业调整振兴政策，建立大企业服务“直通车”制度，实施兰州石化550万吨常减压装置、蓝星公司“1318”、兰州燃化集团液化天然气等重点项目，实现规模以上工业增加值308.17亿元，增长9.83%。积极落实国家“家电下乡”、“汽车下乡”等扩大消费政策，加快实施城区大型批发市场外迁工程，努力营造良好的消费环境，市场物价保持稳定，城乡消费市场活跃，实现社会消费品零售总额469．77亿元，增长18．92%。居民消费价格指数为99.6%。加强旅游业发展规划指导，实现旅游收入37.7亿元，增长22%。抓住兰州被列为全省统筹城乡发展试点市的机遇，加大农业投入，调整农业经济结构，大力发展现代农业，秦王川综合开发、大型泵站更新改造、设施农业示范基地、小城镇和新农村建设加快实施，农业生产规模化、标准化水平明显提高，推广全膜双垄栽培面积38.5万亩，粮食产量和蔬菜产量分别达到38.79万吨和186.66万吨，实现农业增加值30亿元，增长6％。财税金融运行平稳，完成地区性财政收入254.8亿元，增长71.27%；地方财政总收入94.47亿元，增长16.62%：金融机构各项存款余额、贷款余额和居民储蓄存款余额分别增长21.56%、32.03%和20.16%。城市居民人均可支配收入达到12760.66元，增长9.28%；农民人均纯收入突破4000元，增长14%，是近年来增幅最高的一年。

　　(二)项目建设取得明显成效。紧紧抓住国家扩大内需和投资向西部地区倾斜的政策契机，积极做好重大项目建设及论证、争取和储备工作，全市集中实施了100项重大项目，完成投资181.7亿元，是近年来项目建设涉及领域较广、资金到位率较高的一年。争取国家扩大内需项目288项，落实中央投资16.56亿元，开工建设260项、竣工105项。全力支持中央和省属在兰项目建设，积极搞好规划、征地、拆迁、安置等协调服务，兰州铁路枢纽、兰渝铁路、甘肃会展中心等重大项目顺利实施，510所航天科技园、国家石油储备兰州基地、5个国家质检中心、兰州汽车城、液化天然气装备制造基地等项目开工建设，重离子治癌及大科学城项目前期工作进展良好。园区建设步伐加快，高新区“一区多园”发展格局初步形成，实现生产总值120亿元，增长29.17%；经济区“区区合一”优势逐步发挥，实现生产总值59亿元，增长18%。积极开展招商引资工作，组织了赴长三角、环渤海、闽赣渝、香港等招商考察活动。参与举办了第十五届兰洽会。全年签约各类国内合同项目33l项，引进资金到位141.38亿元。

　　(三)城市建设管理水平进一步提升。全面开展了新一轮土地利用和城市总体规划修编及第一版城乡统筹总体规划编制工作，完成城市控详规划和专项规划设计12项，规划在城市建设中的先导作用得到较好发挥。继续推进城市基础设施建设，大砂坪北出口改造、和定公路、城市景观亮化二期等项目建成使用，庙滩子地区整体改造、亚行贷款城市交通、雁滩南河道综合治理三期等项目加快建设，移山造地一期等项目启动实施，城市轨道交通项目前期工作有序推进，特别是多方面积极创造条件，开工建设了南山路工程。完善城市综合管理，出台了投资项目评审、廉租住房保障、城市燃气、旅游行业、城市房屋租赁等管理办法。加大城市环境集中整治力度，健全数字化城市管理系统，强化城市管理绩效考核，市容市貌进一步改观。多渠道筹措城市建设资金，与国家开发银行甘肃分行等金融机构签订600亿元的长期合作协议，成功发行15亿元城投企业债券，实现土地经营收益23.22亿元。

　　(四)节能减排和环境建设积极推进。编制完成全市循环经济发展规划，确定了企业、园区、社会三个层面发展循环经济的工作重点。西固区循环经济试点工作稳步实施。兰州石化、兰铝等大企业资源综合利用、范家坪热电厂建设等节能减排重点项目进展良好。完成125台燃煤锅炉清洁能源改造，综合整治1049家餐饮企业，西热东输新增供热面积112万平方米。通过完善项目节能评估审查政策，有效控制了高耗能、高污染行业过快增长。继续推进城区空气污染治理，空气质量优良天数达到64.7%，污染综合指数下降O.23。城区污水全收集全处理工程完成89公里主管网敷设任务，启动实施西固、盐场、雁儿湾3个污水处理项目。城市垃圾无害化处理工作逐步加强，规划实施的垃圾处理场建成2个、在建3个、完成前期工作4个。全市单位生产总值能耗下降5%，单位工业增加值能耗下降6%以上，二氧化硫和化学需氧量分别下降2.1%和6%，兰州荣获“中国节能减排20佳城市”称号。

　　(五)民生和各项社会事业协调发展。就业工作进一步加强，城镇新增就业5.2万人，高校毕业生就业率82.9%，输转城乡劳动力3l万人次，城镇登记失业率为3.1%。各项社会保障制度逐步完善，提高城市低保标准10%，五区由每人每月230元增加到253元，三县由173元增加到190元；农村低保标准由每人每年685元增加到不低于728元。启动了新型农村社会养老保险试点工作。重视解决城乡困难群众住房问题，174万平方米经济适用住房和48万平方米廉租住房抓紧建设，为7797户群众发放廉租住房补贴2267万元。制定了市属企业危旧房改造整体规划，开工建设70万平方米。农村危旧房改造投资18．66亿元，改造农户3.1万多户。为民兴办的15项20件实事全面完成，特别是解决了11.5万人饮水安全问题，新建农村公路1058公里、客运站150个，建成生态家园沼气池10580户，农村生产生活条件逐步改善。创新型城市建设加快推进，兰州被确定为国家石化新材料产业基地，入选首批国家创新型试点城市，获得“国家知识产权工作示范城市”称号。出台实施“教育满意行动计划”，调整了近郊四区教育布局结构，完成城乡中小学危房改造203所，建成农村寄宿制学校40所。实施了“八办”红色旅游改造项目。新建12个乡镇综合文化站和300个农家书屋。建成120条全民健身路径。举办了市第六届运动会。改扩建23个乡镇卫生院，建成11个社区卫生服务中心和90个村卫生室。完成34个基层司法所建设。“五城联创”工作取得积极进展，启动了新一轮全国文明城市创建工作。举办了庆祝新中国成立和兰州解放60周年大型系列文化活动，激发了广大干部群众热爱兰州、干事创业的热情。民族宗教、广播电视、人口和计划生育、信访、统计、地震、人防、气象、外事、地方志、残疾人等各项工作都取得了新的成效。

　　(六)科学发展的体制机制逐步完善。继续深化国有企业改革，完成24户企业资产重组，建立了国有资产监管体系，健全了现代企业管理制度。全面启动统筹城乡发展试点工作，确定了2个县区、10个重点小城镇、61个城中村和新一轮新农村建设三个层次的试点布局，统筹城乡发展整体规划和相关配套政策加快制定。集体林权制度改革全面启动，试点工作进展顺利。积极探索农村土地流转使用制度，部分县区建立了土地流转平台。财政体制改革逐步深化，市级预算单位全部实现国库集中支付，项目库改革和公务卡结算试点全面展开，皋兰县纳入“省直管县”改革试点范围。积极推进地方金融机构改革，重组成立兰州银行股份有限公司，上市融资步伐加快。吸引金融企业在兰设立分支机构，中信银行兰州分行开业运营。启动市级政府机构改革，机构整合、职能调整、人员分流等工作有序进行。事业单位改革稳步推进，基本完成首次岗位设置管理工作。全市义务教育学校实行了绩效工资制度。

　　(七)各项社会管理不断加强和改进。认真落实维稳工作责任制，深入推进社会治安综合治理，进一步完善城乡治安技防网络，加快推进打防控体系建设，依法严厉打击各类刑事犯罪，社会治安持续稳定，人民群众的安全感普遍提高。高度重视甲型H1N1流感防控工作，防止了疫情传播和扩散。开展食品药品十大专项集中整治活动，建立网格化日常监管机制和监督信息公示制度，解决了一批社会关注的饮食和用药安全问题。加强安全生产工作，层层落实责任制，深入开展重点领域的事故隐患专项整治，各类事故起数、致伤人数、直接经济损失有所下降。规范和改进应急管理工作，各级政府应急机构逐步建立，修订完善了市级应急预案体系，妥善处置九州地质灾害、盐什公路山体滑坡等突发事件125项。针对地质灾害事件中暴露出来的突出问题，全面开展地质灾害隐患排查，完成城市重大地质灾害防治专项规划，投资4000多万元对地质灾害险情和灾情采取综合治理措施，向国家申报了地质灾害综合防治项目，为全面防治地质灾害、消除城市安全隐患创造了条件。

　　(八)政府服务水平有了新的提高。按照中央和省、市委的统一部署和要求，着眼于建设人民满意的政府，认真开展深入学习实践科学发展观活动，着力转变不适应科学发展要求的思想观念，群众对市政府工作评议满意率达到97%。坚持依法行政，自觉接受市人大依法监督和市政协民主监督，办结人大代表议案建议223件、政协委员提案738件。政府立法工作进一步加强，制定和修订地方性法规2部、政府规章7部。完善政府规范性文件制定程序，对关系经济社会发展和群众切身利益的重大事项全面推行政务公开，提高了政府工作的透明度。健全“一站式”集中审批制度，29个部门的416项行政审批事项在政务大厅统一办理，限时办结率达到99．97%。深入开展“机关作风建设年”活动，着力解决突出问题，政风行风明显好转。强化审计监督工作，加大重点行业、重大事项和专项资金审计检查力度，促进了财政财务的规范运行。大力倡导和践行勤俭办事，党政机关公务接待费削减10%，车辆购置运行费降低15%，因公出国经费压缩20%，全年节约各类经费2125万元。

　　各位代表，过去的一年，在各种困难和矛盾比较多的情况下，全市经济社会发展取得的成绩来之不易。这是省委、省政府和市委正确领导的结果，是市人大、市政协监督支持的结果，也是全市各族人民创业实干、共同努力的结果。我们深深感到，现阶段兰州要实现又好又快的发展，必须紧紧围绕发展这个第一要务，注重发挥全市各方面的积极性和主动性，凝心聚力破难题，全力以赴促发展，始终依靠全市人民的智慧和力量建设兰州、发展兰州。在这里，我代表市人民政府，向全市各族人民，各位人大代表和政协委员，各民主党派、工商联、人民团体和无党派人士，离退休老同志和社会各界人士，驻兰解放军指战员、武警官兵和公安干警，以及多年来关心支持兰州现代化建设事业的同志们、朋友们表示衷心的感谢!

　　在肯定成绩的同时，我们也清醒地看到，当前经济社会发展和政府工作还面临诸多矛盾和问题。主要是：在日趋激烈的区域发展竞争中，新的经济增长点不明显，综合实力还不强；科技资源优势发挥不充分，传统产业所占比重较高，高新技术和现代服务业发展不快，现有经济结构对转变发展方式的制约较大；城市基础设施仍较薄弱，生态文明建设任务艰巨，大气污染治理、交通管理亟需进一步加强；“大城市”与“大农村”并存，城乡居民收入差距大，贫困人口量大面广，统筹城乡发展难度较大；地方可支配财力有限，市场化融资渠道单一，建设发展的资金比较紧缺；城市建设用地矛盾突出，建成区面积难以承载开发、建设和发展的需要，拓展城市空间十分迫切；政府职能转变不够到位，公共服务有待加强。对于这些问题，我们将高度重视，采取积极有效的措施，认真加以研究解决。

　　二、2010年工作的总体要求和目标任务

　　今年是实施“十一五”规划的最后一年。做好今年的工作，对于保持全市经济社会持续较快发展，全面完成“十一五”规划目标任务，为“十二五”时期的发展打好基础，加快全面建设小康社会进程具有重要意义。

　　政府工作的总体要求是：以党的十七大、十七届三中、四中全会精神为指导，全面贯彻科学发展观，深入落实省上区域发展战略和市委“1355”总体发展思路，按照“打好六大战役、实现六个突破”的工作部署，着力调整经济结构和转变发展方式，着力加强基础设施建设，着力加快城乡一体化发展，着力推进“兰白都市经济圈”和“兰北新区”建设，着力保障和改善民生，着力创新体制机制和扩大开放，着力转变政府职能和提高行政效能，努力实现全市经济社会加快发展、率先发展、科学发展。

　　经济社会发展的主要预期目标是：生产总值增长11%；规模以上工业增加值增长12%；财政收入增幅高于生产总值增长水平；全社会固定资产投资增长20%；社会消费品零售总额增长18%；城市居民人均可支配收入增长10%，农民人均纯收入增长12.5%；居民消费价格指数103.5%；城镇新增就业5万人，城镇登记失业率控制在4.6%以内；人口自然增长率控制在5.5‰以内；单位生产总值能耗降低4.8%以上。

　　以上预期目标，把握了国内外宏观经济趋向和中央、省上的政策要求，充分考虑了全面完成“十一五”规划目标任务的可行性，体现了全市广大干部群众加快发展的强烈愿望，经过努力是能够实现的。

　　当前，我市发展的内外环境仍然比较复杂，全球金融危机的影响尚未完全消除，经济发展的不可预见因素力调整经济结构和转变发展方式，着力加强基础设施建设，着力加快城乡一体化发展，着力推进“兰白都市经济圈”和“兰北新区”建设，着力保障和改善民生，着力创新体制机制和扩大开放，着力转变政府职能和提高行政效能，努力实现全市经济社会加快发展、率先发展、科学发展。

　　经济社会发展的主要预期目标是：生产总值增长11%；规模以上工业增加值增长12%；财政收入增幅高于生产总值增长水平；全社会固定资产投资增长20%；社会消费品零售总额增长18%；城市居民人均可支配收入增长10%，农民人均纯收入增长12.5%；居民消费价格指数103.5%；城镇新增就业5万人，城镇登记失业率控制在4.6%以内；人口自然增长率控制在5.5‰以内；单位生产总值能耗降低4.8%以上。

　　以上预期目标，把握了国内外宏观经济趋向和中央、省上的政策要求，充分考虑了全面完成“十一五”规划目标任务的可行性，体现了全市广大干部群众加快发展的强烈愿望，经过努力是能够实现的。

　　当前，我市发展的内外环境仍然比较复杂，全球金融危机的影响尚未完全消除，经济发展的不可预见因素还较多，但我们也面临一些新的机遇和条件。一是国家保持宏观经济政策的连续性和稳定性，进一步加大西部大开发战略实施力度，支持西部地区生态环境、基础设施、农业农村和社会事业建设，有利于我们争取项目、扩大投资。二是国务院已经批复甘肃建设循环经济示范区，还将出台支持甘肃加快经济社会发展的政策，兰州将会得到国家更多的支持，形成新一轮开发建设的良好环境。三是在国家有关部委和国际组织的主导下，新亚欧大陆桥建设持续推进，从连云港经兰州到中亚、东欧的交通大通道进一步畅通，将为兰州的开放开发创造新的平台。四是省上实施新的区域发展战略，全面规划建设“兰白都市经济圈”，为兰州长远发展提供了机遇，增添了新的活力。五是省上确定兰州先行实施城乡一体化试点，这是我市破解城乡二元矛盾，加快缩小城乡差距的新机遇。六是兰州作为全国九大综合性交通枢纽之一，随着青藏铁路和连接周边城市高速公路建成运营，兰州铁路枢纽、兰渝铁路和兰新铁路第二双线等项目加快实施，城市交通运输和通达能力将得到较大提升，为兰州建设区域性商贸物流中心创造更为有利的条件。

　　我们将紧紧抓住这些机遇和条件，围绕“打好六大战役、实现六个突破”的部署，咬住目标不放松，一张蓝图绘到底，重点抓好以下七个方面的工作：

　　(一)积极推动落实省上区域发展战略，更好发挥中心城市带动作用。深入分析和把握当前区域发展面临的机遇和条件，在全省、全国大格局中谋划兰州的发展，努力增强城市的集聚、辐射和带动能力。

　　一是加快“兰白都市经济圈”规划建设。建立务实高效的协调推进机制，加快编制科学合理的经济圈建设规划，强化皋兰卫星节点城市连接功能，率先推进都市圈基础设施、产业布局、文化旅游、物流发展和公共服务一体化。

　　二是积极构建“兰北新区”。围绕秦王川盆地、黄河北部和榆中盆地三大区域，实施移山造地、基础设施建设和重点功能配套，打造卫星城市、综合性新城区和新型产业园区，拓展城市发展新空间，培植产业发展增长极。

　　三是修编实施新一轮城市总体规划。坚持城市总体规划修编和实施区域发展战略相结合，以黄河为轴、两山为翼，优化提升城关和七里河老城、安宁新城、西固石化城，构筑青白石、沙中、河口南、和平定远四大片区，逐步形成“大兰州”的城市发展格局。

　　四是推进综合性交通枢纽建设。切实履行地方政府协调服务职责，确保兰州铁路枢纽、兰渝铁路、铁路集装箱中心站、兰新铁路第二双线、宝兰客运专线、中川机场改扩建等重大项目顺利实施，争取启动兰州至张掖增建三、四线铁路项目。

　　五是高起点谋划“十二五”发展。结合“十一五”规划完成情况和未来五年发展重点，全面谋划和科学编制“十二五”规划，重点研究城市空间拓展、产业结构调整、重大基础设施建设、区域联动发展、体制机制创新和对外开放等重大问题，不断完善发展思路，为今后一个时期兰州的发展奠定基础。

　　(二)着力调整和优化经济结构，加快转变发展方式。按照“一产抓特色、二产抓延伸、三产抓转型、整体抓提升”的思路，加快产业布局调整和集聚发展，促进三次产业优化升级，提高经济发展的质量和效益。

　　一是大力发展现代农业。紧紧围绕特色做文章，充分发挥比较优势，培育发展适应市场需求、产出效益较高的特色产业。坚持因地制宜，区分山旱、川水、城郊三大区域，优化农业产业布局，发挥龙头企业和农民专业合作组织作用，加快培育优势农产品生产基地和特色产业带，集中连片发展高效设施农业，扩大高原夏菜、玫瑰、百合等特色农产品生产规模和品牌影响力。新增设施农业面积6000亩，蔬菜种植面积稳定在75万亩，全膜双垄栽培面积达到45万亩。

　　二是加快发展工业经济。坚持把改造提升传统产业与培育壮大新兴产业结合起来，以实施100项重点工业项目为抓手，延伸传统产业链条，大力发展新材料、新能源、先进制造、航空航天装备和生物医药等新兴产业。强化园区建设和功能配套，抓好高新区彭家坪装备制造业生态园区、经济区整区开发、石化科技产业基地、空港循环经济产业园、金川科技产业园建设，引导企业向园区集聚，产业向基地集中，高新区、经济区生产总值分别达到150亿元和70.8亿元。落实《甘肃省循环经济总体规划》，建设以石油化工、有色冶金为主的兰白循环经济基地。加快发展低碳经济，淘汰落后产能。

　　三是提升现代服务业发展水平。积极打造区域性商贸物流中心，制定实施现代物流、商务会展等发展规划，优先培育壮大现代服务业和生产型服务业，着力发展物流产业和新型服务业态。合理规划布局大型市场，推进东部生活资料物流基地、西部生产资料和陆港物流园区、再生资源产业园、兰州汽车城等重点项目建设，促进商品市场集约发展。加快区域性金融中心建设，发挥交通枢纽及能源和有色冶金基地优势，积极向国家争取设立兰州有色金属和能源期货交易所。完善社区便民服务网络和农村市场体系，满足城乡群众多层次的消费需求。

　　四是努力打造区域旅游集散中心。全面实施旅游业发展规划，整合办好富有兰州特色的精品节会，着力发展以黄河文化为代表的品牌旅游，加快把旅游业培育成新型支柱产业，全年旅游接待人数增长18%，旅游收入增长20%。

　　五是大力发展非公有制经济。营造创业文化，推进全民创业，培育非公有制重点骨干企业，建设8个中小企业创业孵化基地，新增3户年销售收入上3亿元的工业企业，20户科技成长型企业，220户年销售收入上千万元的中小企业，力争非公有制经济增加值增长20%以上。

　　(三)全力推进重大项目建设，进一步夯实发展基础。充分发挥投资拉动经济增长的作用，加大统筹管理和协调服务力度，全方位推进重大项目建设。

　　一是争取和落实一批符合国家产业政策的重大项目。按照凝炼、上报、争取、落地、建设的步骤，深入研究国家宏观经济政策和投资导向，积极主动向国家和省上汇报衔接，力争城市重大交通设施建设、地质灾害防治、大气污染防治和黄河兰州段水污染治理等项目获得国家支持。加大对中央扩大内需项目和资金使用情况的监督检查，严格规范项目管理和实施流程，确保资金安全使用、发挥效益。

　　二是集中实施lOO项重大项目。农林水利方面，加快实施秦王川综合开发、大型泵站更新改造等项目。工业能源方面，大力推进吉利轿车扩能改造、佛慈工业园、国家石油储备兰州基地、黄河河口水电站等项目。城市建设方面，抓好南山路、庙滩子地区整体改造、移山造地、黄河大桥、雁滩南河道综合治理、城中村改造等项目。高新技术方面，加快建设5个国家质检中心、重离子治癌及大科学城、510
